# Integrated WGCNA and Network Pharmacology Explore the Potential Mechanisms of D-Limonene in Alleviating Traumatic Brain Injury

**DOI:** 10.3390/ijms27146143

**Published:** 2026-07-09

**Authors:** Hongyan Jiang, Xinyuan Luo, Hengxi Li, Zhiying Guo, Xiao Yan, Ping Li

**Affiliations:** Department of Anatomy and Histology & Embryology, Faculty of Basic Medical Science, Kunming Medical University, Kunming 650500, China; 20241930@kmmu.edu.cn (H.J.); 20240020@kmmu.edu.cn (X.L.); lihengxi@kmmu.edu.cn (H.L.); 20250003@kmmu.edu.cn (Z.G.); 201902040@kmmu.edu.cn (X.Y.)

**Keywords:** D-limonene, blood–brain barrier, network pharmacology, traumatic brain injury, neuroinflammation

## Abstract

D-limonene (D-Lim) is a monocyclic monoterpene and the principal component of citrus essential oils; however, the potential mechanisms underlying its neuroprotective effects in traumatic brain injury (TBI) remain incompletely elucidated. By integrating network pharmacology, weighted gene co-expression network analysis (WGCNA), molecular docking, and experimental validation, this study systematically investigated the potential mechanisms through which D-Lim exerts neuroprotective activity. Database analyses and in vivo experiments showed that the anti-inflammatory and neuroprotective effects produced by D-Lim may be related to the p38 MAPK/NF-κB signaling axis and activation of the PI3K/AKT signaling pathway. Molecular docking and RT-PCR experiments indicated interactions between D-Lim and potential target proteins, including *Icam1*, *Kdr*, and *Dpp4*. HE and Nissl staining demonstrated that D-Lim ameliorated TBI-induced neuronal injury. Moreover, D-Lim had no observable effects on major organs and showed no peripheral toxicity, suggesting its favorable applicability for TBI intervention. Following early intervention with D-Lim, the inflammatory response induced by TBI was attenuated, which may be associated with the activation or modulation of the p38 MAPK, PI3K/AKT, and NF-κB p65 signaling pathways. These results indicate a potential acute protective role for D-Lim under prophylactic or early-intervention conditions, provide insights into TBI intervention, and establish a theoretical basis for potential preventive strategies.

## 1. Introduction

Traumatic brain injury (TBI) is an acquired brain injury, which caused by external mechanical forces, affecting about 70 million patients all over the world each year [1,2]. TBI is a leading cause of death among adolescents worldwide, with particularly high morbidity and mortality in low- and middle-income countries, making it a major global health challenge [3]. TBI not only causes primary structural damage but also acutely alters cerebral metabolism and hemodynamics, triggering cellular dysfunction and subsequent secondary injury [4]. Specifically, primary injury mainly includes localized damage such as cerebral contusion and laceration, skull fracture, and penetrating brain injury, which can directly lead to disruption of the blood–brain barrier (BBB), axonal fiber damage, and neuronal death [5]. On this basis, a series of secondary cascade reactions are induced, mainly including neuroinflammation, mitochondrial dysfunction, lipid peroxidation, oxidative stress, excitotoxicity, and cerebral hypoperfusion [4,6,7]. Following TBI, disruption of the BBB activates resident microglia, leading to the release of harmful factors such as nitric oxide (NO), tumor necrosis factor-alpha (TNF-α), and interleukin-1β (IL-1β), which exacerbate neuronal damage and are detrimental to patient prognosis [8,9]. Among these, neuroinflammation plays a central role of secondary injury following TBI. If left untreated or if intervention is delayed, it can exacerbate the inflammatory cascade and further aggravate neuronal damage [10,11]. However, conventional treatment for TBI mainly includes lowering intracranial pressure, maintaining blood oxygenation, and surgical intervention [12]. Despite some progress having been made in the management of TBI, the mortality and disability rates attributable to TBI remain high. Currently, effective pharmacological treatments for patients with TBI are still extremely limited [6], underscoring the urgent clinical need for the development of therapeutic drugs for TBI.

In China and other regions deeply influenced by Chinese culture, traditional Chinese medicine (TCM), as well as natural products and their pharmacologically active molecules derived from TCM theories, have garnered significant attention in TBI research owing to their multi-target, multi-pathway regulatory characteristics [13]. However, the clinical translation of some candidate natural products is still constrained by pharmacokinetics and the feasibility of administration, such as problems like poor water solubility or low oral bioavailability for compounds like cannabidiol, curcumin, and quercetin, which limit their direct translational potential [14,15,16]. Although resveratrol is well absorbed, its systemic bioavailability is relatively low due to rapid metabolism, and maintaining an effective concentration in the brain remains a challenge [17]. In contrast, D-limonene (D-Lim), the main monoterpenoid component in the volatile oils of citrus plants, has the characteristics of wide availability, strong lipid solubility, and a good safety profile [18,19], and has demonstrated anti-inflammatory, antioxidant, antiapoptotic, and neuroprotective effects in models related to neurodegenerative injuries [20,21]. These features make D-Lim have good priority research value in the multi-link intervention targeting secondary injuries of TBI. However, there is still a lack of systematic research on the role and potential mechanism of D-Lim in TBI. Therefore, this study intends to combine network pharmacology and in vivo experiments for verification, to explore the therapeutic effect and possible mechanism of D-Lim on TBI, and to provide new experimental basis and candidate strategies for the use of natural products in TBI adjunctive intervention.

Among them, D-Lim is widely distributed in foods such as fruits, vegetables, and meat [22,23,24]. Studies have shown that D-Lim possesses potent antitumor, hepatoprotective, immunomodulatory, anti-inflammatory, and antispasmodic properties [25,26,27,28,29]. And it exhibits antidiabetic, antihypertensive, antioxidant, anti-inflammatory, antinociceptive, and anticancer effects in the treatment of diabetes, hypertension, and cancer [30,31,32]. Existing evidence indicates that long-term use of D-Lim, or its combination with honey, can alleviate hyperlipidemia-induced brain injury [33]. In an ischemic stroke model, D-Lim has been confirmed to exert multiple neuroprotective effects by reducing post-ischemic cerebral vasoconstriction through its antioxidant activity, while also inhibiting neuroinflammation and promoting angiogenesis [34]. Moreover, as a key anti-inflammatory component in the volatile oil of grapefruit peel, D-Lim alleviates neuroinflammation in rats after cerebral ischemia–reperfusion by blocking the TLR4/NF-κB pathway [35]. In brain tumors, D-Lim targets and kills tumor cells by inducing endoplasmic reticulum stress and increasing reactive oxygen species (ROS) levels, which cause cell damage and apoptosis [36]. Given the potent pharmacological effects of D-Lim, numerous studies have investigated its role in non-TBI conditions. However, evidence regarding its effects and mechanisms in TBI remains to be further elucidated, which provides a rationale for exploring its therapeutic efficacy following TBI in the present study.

As high-throughput sequencing rapidly advances, bio-network-based integrated analysis strategies have become increasingly mature, providing novel research perspectives and theoretical support for unraveling disease mechanisms and screening potential disease biomarkers [37]. Meanwhile, bioinformatics analysis, as an efficient and promising research approach, has been widely used to screen for significantly differentially expressed genes (DEGs). Among these, weighted gene co-expression network analysis (WGCNA), a common systems biology method, is used to identify disease markers and therapeutic targets [38]. Furthermore, network pharmacology, as an emerging tool in the field of drug discovery, integrates compound screening, target prediction, and pathway enrichment analysis to construct potential drug–target interaction networks, thereby providing critical support for candidate drug screening and mechanistic studies of drug targets [39].

Accordingly, in this study, we systematically screened and evaluated the potential targets of D-Lim in TBI. Combining network pharmacology with WGCNA, we sought to explore the potential molecular mechanisms by which D-Lim exerts its regulatory effects on TBI. Ultimately, this research is expected to offer a theoretical basis and fresh perspectives for advancing clinical strategies in TBI management.

## 2. Results

### 2.1. WGCNA Sample Clustering and Soft-Threshold Screening

**Figure 1:**
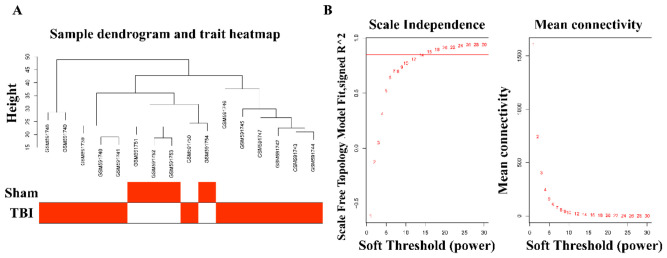
Sample clustering and soft-threshold screening of the GSE24047 dataset. (**A**) Hierarchical clustering dendrogram of the 16 samples in the GSE24047 dataset, including 4 sham samples and 12 TBI samples; no obvious outlier samples were identified. (**B**) Network topology analysis under different soft-thresholding powers (β). The left panel shows the scale-free topology fit index, and the right panel shows mean connectivity.

To construct a stable weighted gene co-expression network, WGCNA was performed on the GSE24047 dataset. This dataset contained 16 samples, including 4 sham samples and 12 TBI samples. Hierarchical clustering analysis was performed for all samples to evaluate overall expression patterns and identify potential outliers. The results showed that the samples clustered well overall, and no obvious outlier samples were detected (Figure 1A), indicating that the data quality met the requirements for subsequent WGCNA. Subsequently, different soft-thresholding powers (β) were screened using the pickSoftThreshold function in the WGCNA package. The optimal soft threshold was determined by comprehensively evaluating the scale-free topology fit index and mean connectivity (Figure 1B). When β increased to 16, the signed R^2^ value of the network first exceeded 0.85, while the mean connectivity tended to stabilize. Therefore, β = 16 was selected as the soft-thresholding power for subsequent co-expression network construction to ensure that the network conformed to scale-free topology.

### 2.2. Construction of Co-Expression Modules and Module-Trait Correlation Analysis

**Figure 2:**
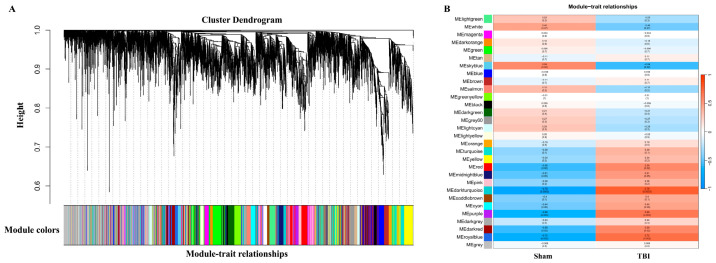
Construction of co-expression modules and module-trait correlation analysis of the GSE24047 dataset. (**A**) Module-trait correlation heatmap. Correlations between different color modules and the Sham and TBI groups are shown by color: red indicates a positive correlation, blue indicates a negative correlation, and darker colors indicate stronger correlations. (**B**) Gene clustering tree and module assignment results. Genes were hierarchically clustered based on the topological overlap matrix (TOM), and different colors represent different co-expression modules.

Based on the optimal soft threshold β = 16, a weighted gene co-expression network was constructed for the GSE24047 dataset, and multiple co-expression modules represented by different colors were identified (Figure 2A). Further analysis of the correlations between each module and the TBI phenotype (Figure 2B), showed that some modules were significantly positively or negatively correlated with the brain injury phenotype, suggesting that these modules may participate in biological processes involved in the occurrence and progression of brain injury.

### 2.3. Correlation Analysis Between GS and MM in Key Modules

**Figure 3:**
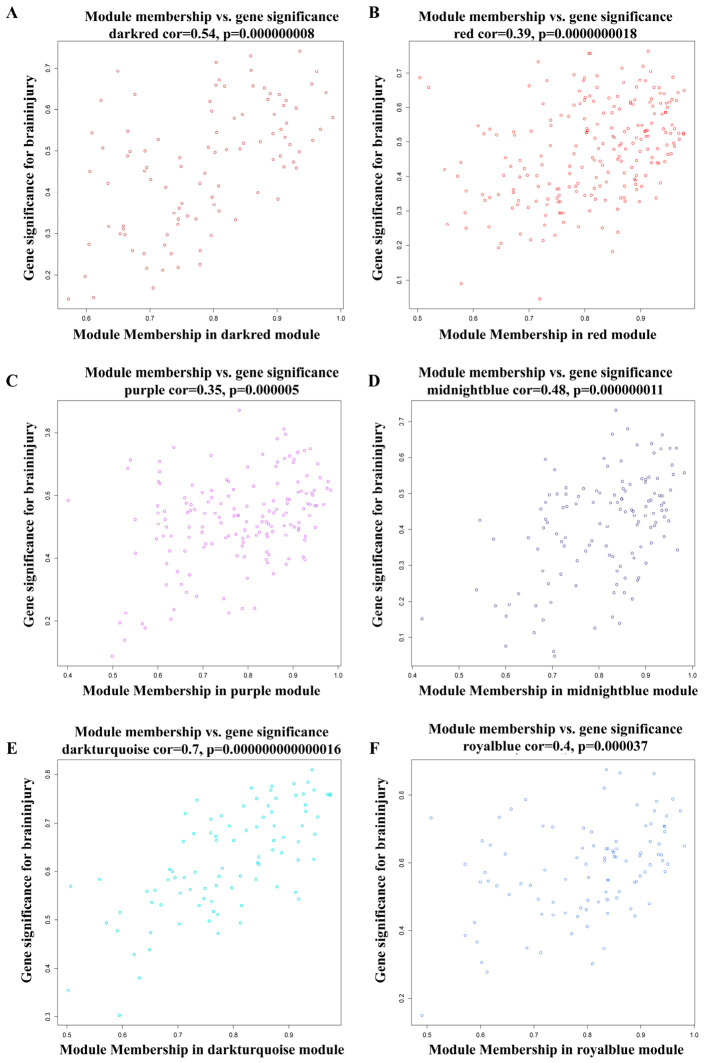
Correlation analysis between gene significance and module membership in key modules. (**A**) GS-MM correlation analysis in the dark red module; (**B**) red module; (**C**) purple module; (**D**) midnight blue module; (**E**) dark turquoise module; and (**F**) royal blue module.

To further screen key modules associated with TBI, correlation analysis between gene significance (GS) and module membership (MM) was performed for significantly positively correlated modules. The results showed that GS and MM were significantly positively correlated in the dark red, red, purple, midnight blue, dark turquoise, and royal blue modules (Figure 3A–F). These findings suggest that genes within these modules were not only highly consistent with the module expression patterns but also closely associated with the brain injury phenotype, and may therefore play important roles in the occurrence and progression of TBI.

### 2.4. Intersection Between Potential Targets and Key Module Genes and Construction of the PPI Network

**Figure 4:**
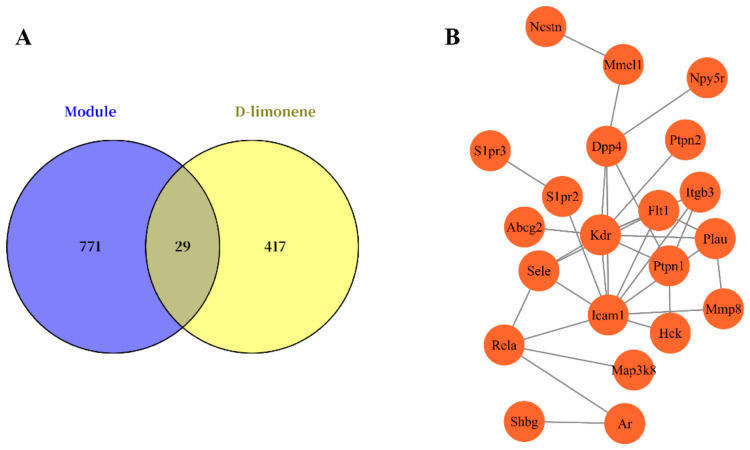
Intersection between predicted targets of D-Lim and key module genes and construction of the PPI network. (**A**) Venn diagram of predicted D-Lim potential targets and WGCNA-derived key module genes. (**B**) PPI network of the 29 potential target genes. Nodes represent proteins, and edges represent protein–protein interaction.

Potential targets of D-Lim were predicted using the Swiss Target Prediction and TargetNet databases and then intersected with the key module genes screened by WGCNA. A total of 29 potential target genes were obtained (Figure 4A). Subsequently, a PPI network was constructed for the intersecting genes, and the results showed interactions among these genes (Figure 4B, Table 1).

### 2.5. GO Functional and KEGG Enrichment Analyses of Intersecting Gene Targets

**Figure 5:**
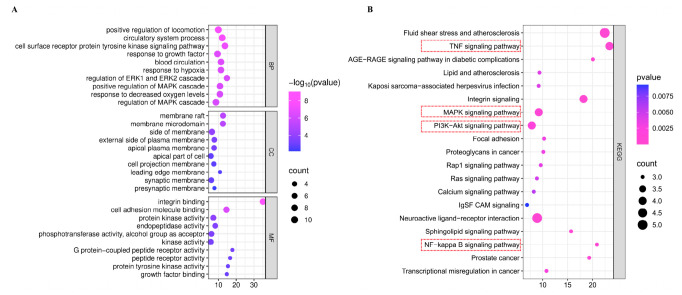
GO and KEGG enrichment analyses of intersecting genes. (**A**) Visualization of GO enrichment analysis. (**B**) Visualization of KEGG enrichment analysis.

GO functional enrichment analysis mainly included three categories: biological process (BP), molecular function (MF), and cellular component (CC). BP terms included response to hypoxia, regulation of blood circulation, and inflammatory response. MF terms involved integrin binding, cell adhesion molecule binding, protein kinase activity, endopeptidase activity, phosphotransferase activity, alcohol group as acceptor, kinase activity, G protein-coupled peptide receptor activity, peptide receptor activity, protein tyrosine kinase activity, and growth factor activity. CC terms included membrane raft, membrane microdomain, side of membrane, synaptic membrane, and presynaptic membrane. The top 10 GO enrichment terms were displayed as a bar chart (Figure 5A). Further KEGG pathway enrichment analysis showed that the 29 potential targets were mainly enriched in inflammation-related pathways, including the TNF signaling pathway, NF-κB p65 signaling pathway, p38 MAPK signaling pathway, and PI3K-AKT signaling pathway (Figure 5B). Among these pathways, the TNF, NF-κB p65, and p38 MAPK signaling pathways are closely related to the release of inflammatory factors, activation of immune cells, and neuroinflammatory responses, whereas the PI3K-AKT signaling pathway participates in regulation of the inflammatory microenvironment and cellular stress responses. These results suggest that D-Lim may attenuate neuroinflammation after TBI and exert neuroprotective effects by regulating inflammation-related signaling pathways.

### 2.6. Screening of Potential Genes in the PPI Network

**Figure 6:**
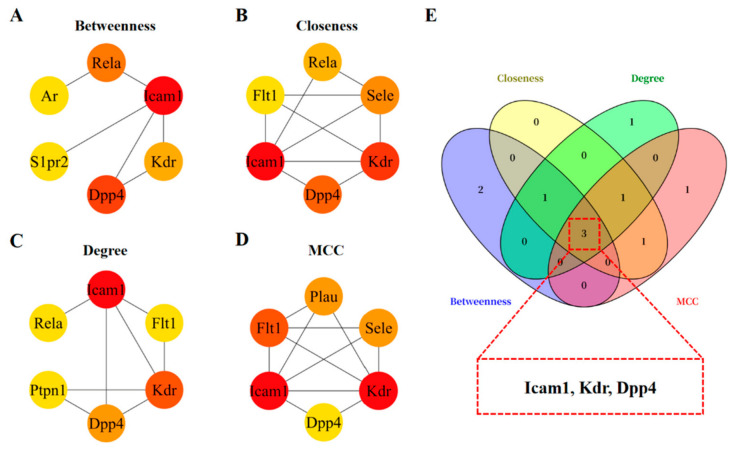
Screening of hub genes in the PPI network. (**A**–**D**) Potential genes in the PPI network were screened using four topological algorithms: MCC, Betweenness, Closeness, and Degree. Node color from yellow to red indicates increasing node importance. (**E**) Venn diagram analysis of the screening results from the four topological algorithms.

To further screen potential genes involved in the effects of D-Lim on TBI, key nodes in the PPI network were analyzed using four topological algorithms: MCC, Betweenness, Closeness, and Degree (Figure 6A–D). The results showed partial overlap among the genes identified by different algorithms. By taking the intersection of the four algorithms, three candidate genes were obtained: *Icam1*, *Kdr*, and *Dpp4* (Figure 6E, Table 2). These findings suggest that these genes may play important roles in the regulation of TBI by D-Lim.

To validate the stability of the hub genes identified in the previous screening using an independent dataset, we further included the GEO transcriptomic dataset GSE80174 and externally validated the expression levels of *Icam1*, *Kdr*, and *Dpp4* between the Sham and TBI groups. The results showed that all three genes were expressed at higher levels in the TBI group than in the Sham group (Figure S1). Overall, the validation results from GSE80174 were consistent with the direction of the initial screening results, further strengthening the evidence linking *Icam1*, *Kdr*, and *Dpp4* to the pathological process of TBI.

### 2.7. Molecular Docking Between D-Lim and the Hub Genes Icam1, Kdr, and Dpp4

**Figure 7:**
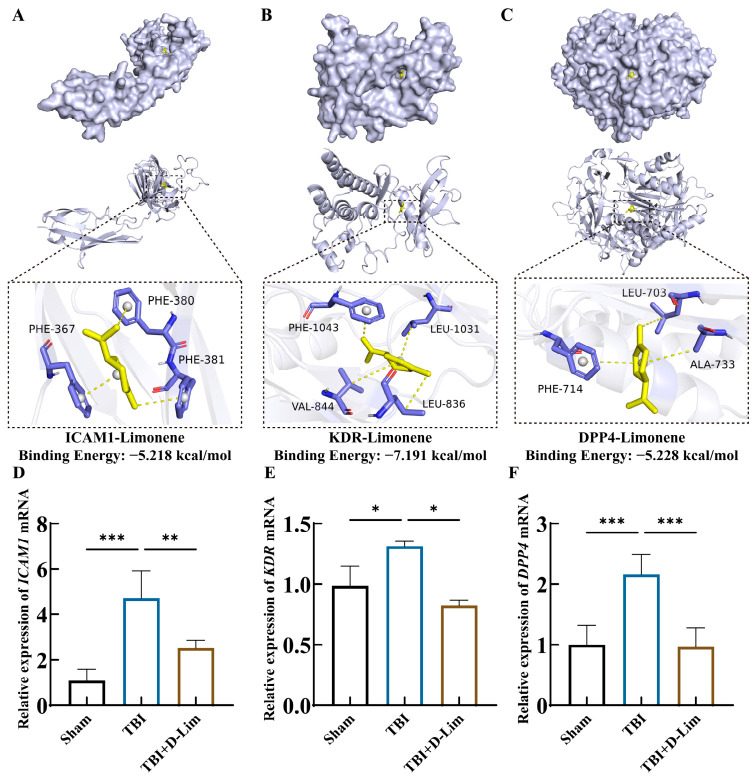
Exploration of the relationship between D-Lim and *Icam1*, *Kdr*, and *Dpp4*. (**A**–**C**) The molecular docking analysis initially discovered the potential binding relationships between D-Lim and *Icam1*, *Kdr*, and *Dpp4*. (**D**–**F**) RT-PCR detection of *Icam1*, *Kdr*, and *Dpp4* mRNA expression after TBI. * *p* < 0.05, ** *p* < 0.01, *** *p* < 0.001. For RT-PCR, each sample was analyzed repeatedly (n = 3, N = 3 rats). The data were presented as mean ± SEM. The data were analyzed using analysis of variance (ANOVA), followed by post hoc tests.

To further explore the potential interactions between D-Lim and the potential targets, molecular docking analysis was performed for *Icam1*, *Kdr*, and *Dpp4*. In general, a binding energy below 0 kcal·mol^−1^ indicates that the receptor and ligand can bind spontaneously without external energy; a binding energy below −5 kcal·mol^−1^ indicates favorable binding; and a binding energy below −7.2 kcal·mol^−1^ indicates strong binding with high affinity [40]. The molecular docking results showed that the binding energies of D-Lim with *Dpp4*, *Icam1*, and *Kdr* were −5.228, −5.218, and −7.191 kcal·mol^−1^. Respectively, indicating it is indicated that there is a potential binding ability between *Icam1*, *Dpp4*, *Kdr* and D-Lim (Table 3). The visualization results indicate that the binding of D-Lim to *Icam1*, *kdr*, and *Dpp4* may mainly rely on non-covalent interactions, such as hydrophobic interactions and van der Waals forces (Figure 7A–C). In addition, RT-qPCR was used to detect the mRNA expression of *Icam1*, *Kdr*, and *Dpp4*. The results showed that *Icam1*, *Kdr*, and *Dpp4* mRNA expression was significantly increased after TBI, whereas this upregulation was reversed after D-Lim intervention (Figure 7D–F). These results suggest that *Icam1*, *Kdr*, and *Dpp4* may be potential targets through which D-Lim exerts anti-inflammatory and neuroprotective effects.

To further investigate the potential interactions between D-Lim and *Icam1*, *Kdr*, and *Dpp4*, we performed molecular dynamics (MD) simulations. The RMSD curve showed rapid conformational relaxation at the early stage of the simulations, followed by stabilization, indicating that the D-Lim-protein systems reached dynamic equilibrium (Figure S2A). Consistently, the radius of gyration (Rg) decreased and then plateaued, suggesting that D-Lim molecules shifted from a dispersed distribution to locally aggregated states on the protein surface (Figure S2B). LJ-SR energy analysis showed a gradual decrease to stable negative values, supporting the formation of favorable short-range van der Waals contacts and hydrophobic interactions between D-Lim and the surfaces of *Dpp4*, *Icam1*, and *Kdr* (Figure S2C). Structural snapshots at 0, 25, 50, 75, and 100 ns (extended to 150 ns for *Icam1*) further showed progressive enrichment of D-Lim molecules at hydrophobic surface regions. This aggregation was particularly evident in the *Dpp4* and *Kdr* systems, where relatively stable local adsorption layers formed. *Icam1* exhibited a similar enrichment pattern after extended simulation (Figure S2D–F).

### 2.8. D-Lim Shows Good In Vivo Biosafety and No Acute Toxicity

**Figure 8:**
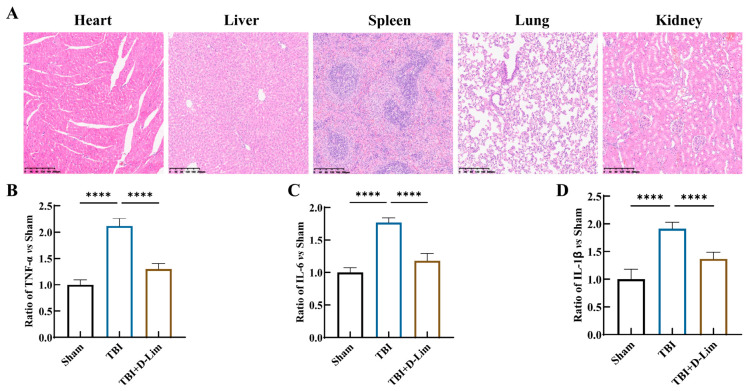
D-Lim has no toxic side effects in rats. (**A**) HE staining (×10, Bar = 200 µm). (**B**–**D**) ELISA detection of TNF-α, IL-1β, and IL-6 levels in peripheral blood, **** *p* < 0.0001. For HE staining and ELISA, each sample was analyzed repeatedly (n = 3, N = 3 rats). The data were presented as mean ± SEM. The data were analyzed using analysis of variance (ANOVA), followed by post hoc tests.

To evaluate the in vivo safety of D-Lim, rats were intraperitoneally injected with D-Lim (10 mg/kg). There were no abnormal manifestations or deaths were observed during the 24 h observation period. HE staining of major organs, including the liver, heart, spleen, lung, and kidney, in the D-Lim intervention group showed no obvious pathological changes (Figure 8A), indicating that D-Lim had no acute toxicity. ELISA analysis of peripheral blood samples showed that the levels of TNF-α, IL-1β, and IL-6 were significantly increased after TBI compared with Sham group. Compared with TBI group, D-Lim intervention significantly reduced the levels of TNF-α, IL-1β, and IL-6 (Figure 8B–D). Our findings, derived from the present experimental setting, indicate that D-Lim not only lacks discernible organ toxicity or systemic adverse effects but also markedly suppresses the expression of TNF-α, IL-1β, and IL-6 in peripheral blood after TBI. Collectively, these data attest to the safety of D-Lim and establish a rationale for subsequent experimental validation.

### 2.9. Potential Effects of D-Lim on the PI3K/AKT, p38 MAPK, and NF-κB p65 Signaling Pathways After TBI

**Figure 9:**
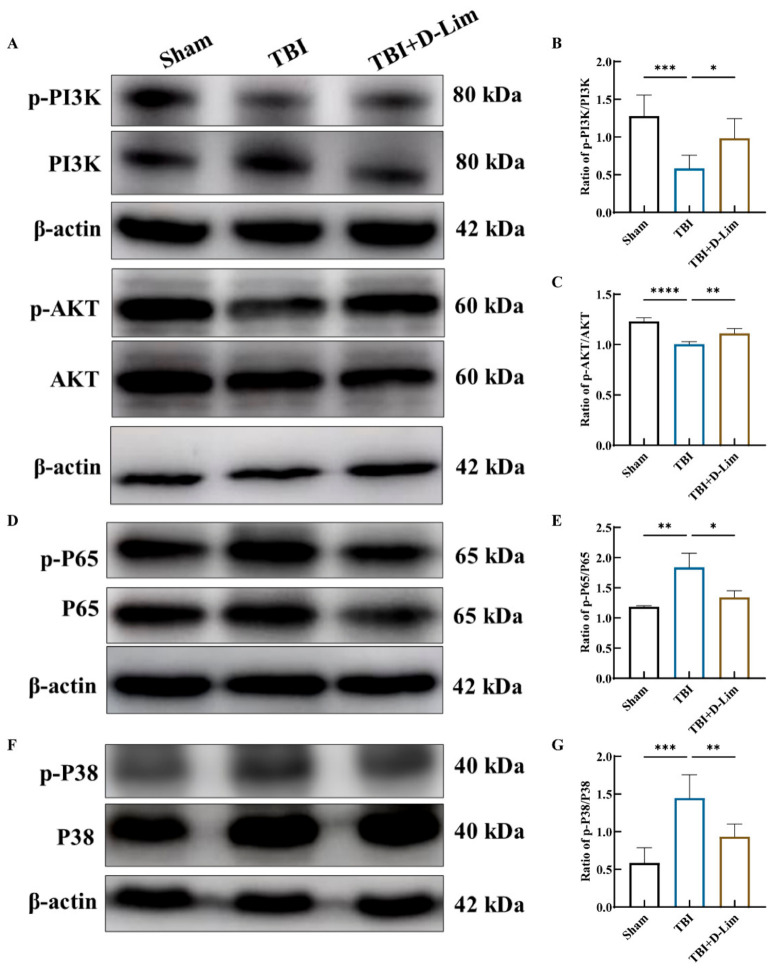
Potential effects of D-Lim on the PI3K/AKT, p38 MAPK, and NF-κB p65 pathways after TBI in rats. (**A**–**C**) Western blot analysis showed that PI3K/AKT phosphorylation was decreased in the TBI group and was significantly increased after D-Lim intervention. (**D**–**G**) Western blot analysis showed that p38 MAPK and NF-κB p65 phosphorylation was increased in the TBI group and was significantly reduced after D-Lim intervention. * *p* < 0.05, ** *p* < 0.01, *** *p* < 0.001, **** *p* < 0.0001. For Western blot, each sample was analyzed repeatedly (n = 3, N = 4 rats). The data were analyzed using analysis of variance (ANOVA), followed by post hoc tests.

To further investigate whether D-Lim regulates PI3K/AKT, p38 MAPK, and NF-κB p65 pathways after TBI, Western blot analysis was used to monitor and evaluate changes in the expression of PI3K/AKT, p38 MAPK, and NF-κB p65 following D-Lim intervention after TBI (Figure 9). The results showed that, compared with the Sham group, the phosphorylation levels of PI3K/AKT were decreased in the TBI group, whereas the phosphorylation levels of p38 MAPK and NF-κB p65 were significantly increased (Figure 9A–C). Compared with the TBI group, D-Lim intervention significantly increased, while the phosphorylation levels of PI3K/AKT. In contrast, the phosphorylation levels of p38 MAPK and NF-κB p65 were decreased (Figure 9D–G). These findings indicate that the early use of D-Lim intervention after traumatic brain injury is, to a certain extent, beneficial in suppressing the inflammatory response. It is speculated that the anti-inflammatory effect of D-Lim may be related to the regulation of the PI3K/AKT, p38 MAPK, and NF-κB p65 pathways.

### 2.10. D-Lim Inhibit the Inflammatory Response and Increasing Blood Flow to the Brain Tissue

**Figure 10:**
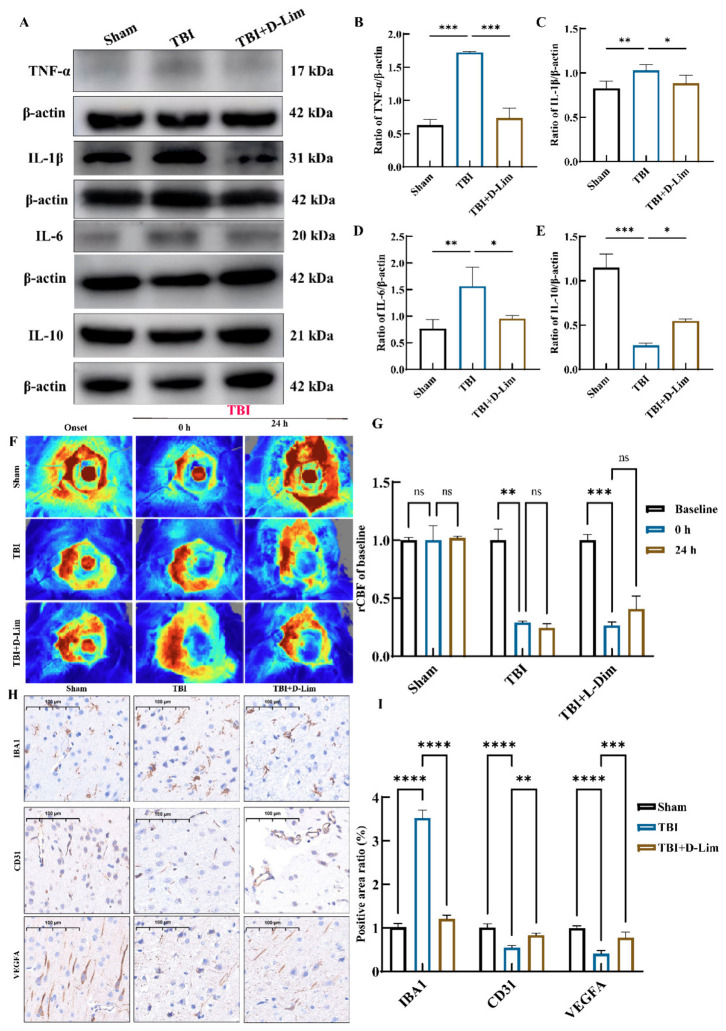
D-Lim attenuates post-TBI inflammation, promotes angiogenesis, and improves cerebral blood flow in injured cortex of rats. (**A**–**E**) Western blot analysis showed that protein expression of TNF-α, IL-1β, and IL-6 was increased and IL-10 expression was decreased in the TBI group, and these changes were significantly reversed after D-Lim treatment. (**F**,**G**) Laser speckle imaging was used to observe changes in relative cerebral blood flow in the injured brain tissue. (**H**,**I**) Immunohistochemical staining showing the expression of IBA1, CD31, and VEGFA in each experimental group (×20, bar = 100 µm). ^ns^ *p* > 0.05, * *p* < 0.05, ** *p* < 0.01, *** *p* < 0.001, **** *p* < 0.0001. For Western blot, each sample was analyzed repeatedly (n = 3, N = 4 rats). For IHC staining, each sample was analyzed repeatedly (n = 3, N = 3 rats). For laser speckle, each sample was analyzed repeatedly (n = 3, N = 3 rats). The data were presented as mean ± SEM. The data were analyzed using analysis of variance (ANOVA), followed by post hoc tests. Two-way repeated-measures ANOVA with Tukey’s post hoc test was used to compare rCBF among groups and across time points.

To further explore the beneficial anti-inflammatory effects of D-Lim after TBI, the expression of related inflammatory factors was examined by Western blotting. Western blot analysis showed that, compared with the Sham group, the expression levels of TNF-α, IL-1β, and IL-6 were significantly upregulated after TBI, whereas IL-10 expression was decreased. Compared with the TBI group, D-Lim intervention decreased the expression of TNF-α, IL-1β, and IL-6 and increased the expression of IL-10 (Figure 10A–E). These results indicate that D-Lim significantly inhibited the protein expression of TNF-α, IL-1β, and IL-6 in the injured cortical tissue after TBI and promoted IL-10 expression, thereby reducing neuroinflammation. In addition, laser speckle imaging showed that, compared with the Sham group, TBI immediately caused a significant reduction in cerebral blood flow at the injured site, and cerebral blood flow remained decreased 24 h after TBI. Compared with the TBI group, cerebral blood flow at the injured site was increased 24 h after D-Lim intervention (Figure 10F,G). The immunohistochemical staining results showed that after TBI, microglia were activated and the expression of CD31 and VEGFA decreased. After D-Lim intervention, the activation of microglial cells was reduced, and the expression of CD31 and VEGFA increased, suggesting that D-Lim can regulate the inflammatory response and participate in the formation of blood vessels as well as the improvement of blood flow in brain tissue (Figure 10H).

### 2.11. D-Lim Improves BBB Permeability, Inhibits Cell Apoptosis, and Exerts Neuroprotective Effects

**Figure 11:**
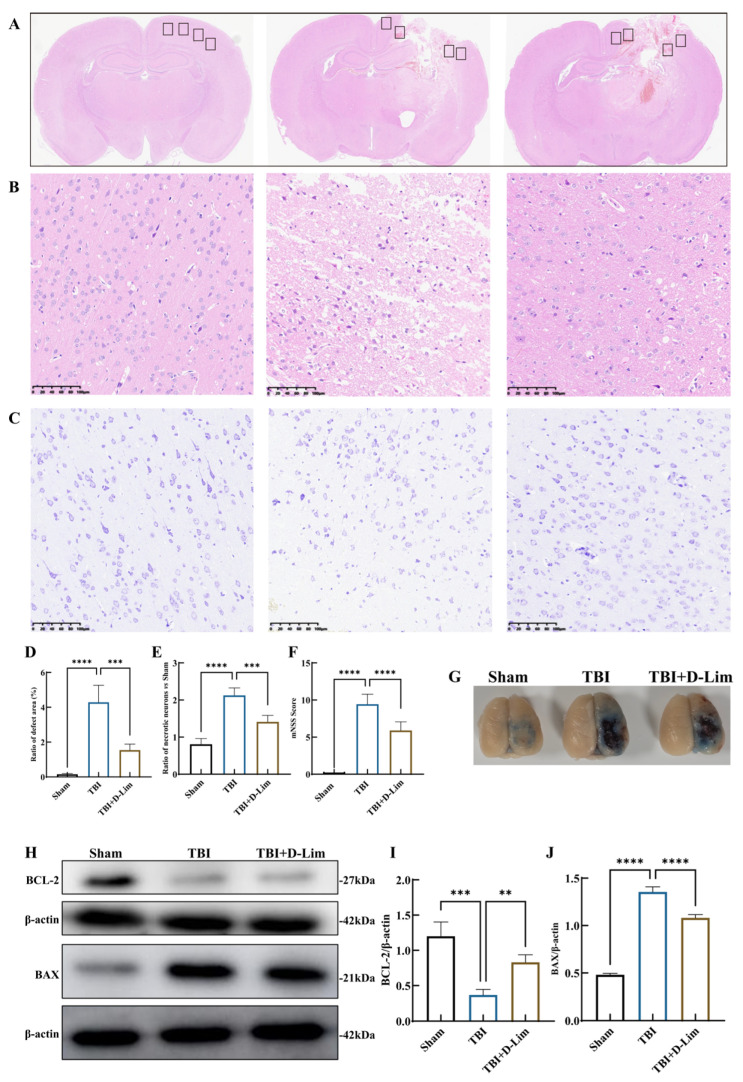
D-Lim attenuates TBI-induced brain injury. (**A**) HE staining images of brain tissues from each experimental group; black boxes denote the primary regions used for pathological evaluation. (**B**) HE staining showing pathological alterations in the injured cortex (×20, bar = 100 µm). (**C**) Nissl staining in the injured cortex (×20, bar = 100 µm). (**D**) Quantification of the brain tissue damage area. (**E**) Neuronal necrosis rate. (**F**) Neurological severity scores (mNSS) assessed at indicated time points. (**G**) Evans blue extravasation assay for blood–brain barrier permeability; upper panel shows macroscopic images, lower panel displays dye penetration into brain parenchyma. (**H**–**J**) Western blot analysis of BCL-2 and BAX protein expression. Data are presented as mean ± SEM. Statistical significance was determined by one-way ANOVA followed by Tukey’s post hoc test. ** *p* < 0.01, *** *p* < 0.001, **** *p* < 0.0001. For Western blot, each sample was analyzed repeatedly (n = 3, N = 4 rats). For mNSS, each group need 10 rats. For HE and Nissl staining, each sample was analyzed repeatedly (n = 3, N = 3 rats). For laser speckle, each sample was analyzed repeatedly (n = 3, N = 3 rats). For Evans blue staining, each sample was analyzed repeatedly (n = 3, N = 3 rats). The data were presented as mean ± SEM. The data were analyzed using analysis of variance (ANOVA), followed by post hoc tests.

HE staining, and Nissl staining showed that TBI induced neuronal injury in the injured cortical region (Figure 11A–C,E). By observing the area of brain tissue damage after TBI through continuous sectioning, the results showed that the brain tissue in the Sham group was intact, while the brain tissue in the TBI group showed damage, which was much more severe than that of the Sham group. Compared with TBI, the brain tissue damage was alleviated after D-Lim intervention (Figure 11D). The mNSS results showed that, compared with the Sham group, scores were significantly increased in the TBI group. Compared with the TBI group, scores were significantly decreased after D-Lim intervention (Figure 11F), suggesting that D-Lim effectively improved neurological deficits induced by TBI in rats. Evans blue staining revealed minimal EB extravasation in the Sham group. TBI significantly disrupted BBB integrity, leading to increased EB leakage and blue staining of brain tissue. However, D-Lim intervention restored BBB function, as evidenced by reduced EB content (Figure 11G). Western blot analysis showed that after TBI, the expression of BCL-2 decreased, while the expression of BAX increased. After D-Lim intervention, the expression of BCL-2 increased, while the expression of BAX decreased (Figure 11H–J). These results indicate that the early use of D-Lim intervention after TBI can inhibit cell apoptosis and have a certain neuroprotective effect.

## 3. Discussion

During its progression, TBI triggers multiple molecular mechanisms that disrupt the BBB, thereby initiating a series of inflammatory cascade reactions and aggravating brain injury [6,41,42,43]. Therefore, controlling the progression of inflammation after TBI is a major therapeutic strategy. However, because of the complexity of its pathogenesis, effective therapeutic drugs remain limited. In recent years, clinical medicine has increasingly focused on natural compounds and medicinal plants because of their fewer side effects, low cost, and favorable therapeutic efficacy [44]. D-Lim, a natural monocyclic monoterpene, exhibits multiple biological activities, including anti-inflammatory, antioxidant, anticancer, antidiabetic, and neuroprotective effects [25,27,29,30,45]. To clarify the regulatory mechanism of D-Lim in TBI, we used WGCNA combined with network pharmacology to predict its potential targets and investigate the possible mechanisms underlying its neuroprotective effects.

In the study, bioinformatics analysis was first performed to identify potential genes after TBI based on the GEO database. Subsequently, potential targets of D-Lim were screened using network pharmacology, and disease-related targets obtained from WGCNA were intersected with D-Lim targets to reveal the complex interactions between the drug and the disease. On this basis, virtual analysis was used to predict the association between D-Lim treatment of TBI and inflammatory responses, with the aim of providing a stronger theoretical basis for its clinical application. The results showed overlapping targets between D-Lim and TBI, supporting the hypothesis that D-Lim may be used for TBI treatment. In addition, GO and KEGG enrichment analyses showed that these overlapping genes were mainly related to inflammatory responses and cell survival, indicating that inflammation plays an important role throughout the pathological process after TBI [46,47]. The attenuation of TBI-induced inflammation after D-Lim treatment suggests that the neuroprotective effect of D-Lim in TBI may be associated with inhibition of inflammatory responses.

Although our bioinformatics screening has revealed candidate genes and pathways relevant to TBI, the findings are subject to certain limitations. Chief among these is the reliance on a single dataset (GSE24047), which was derived from microarray profiling of brain tissues in a rat model of hydraulic impact injury [48]. Consequently, the results may be confounded by dataset-specific variables, such as sample size, experimental time points, array platform, data preprocessing methods, and batch effects. Public transcriptomic datasets are of great value for proposing hypotheses and secondary discoveries, but the results obtained from a single dataset may not fully reflect the biological heterogeneity among species, injury patterns, disease stages, or clinical populations [49]. In this study, we have again verified the hub genes (*Icam1*, *Dpp4*, *Kdr*) in other public transcriptomic datasets (GSE80174). Therefore, the WGCNA results were interpreted as exploratory biological information and were further validated through experiments. Future studies will consider validation using multiple datasets, different animal models, and functional experiments.

Specifically, the GSE24047 dataset was derived from the study by Shojo, in which a moderate fluid percussion injury (FPI) model was established in Sprague-Dawley rats [48]. Brain tissues were collected at 3, 6, 12, and 48 h after injury, and gene expression was measured using the Affymetrix GeneChip Rat Genome 230 2.0 platform (GPL1355). As the present study aimed to identify candidate genes associated with TBI, samples from all post-injury time points (3, 6, 12, and 48 h) were combined as the TBI group and compared with the Sham group, rather than being analyzed as a time series dataset. In addition, we further clarified that both the FPI model used in GSE24047 and the Feeney weight-drop model used in our animal experiments are classical rat models of TBI [50]. Although these two models differ in their specific injury procedures, both can simulate neuroinflammatory responses and secondary pathological changes after TBI, and therefore have good biological relevance and comparability.

After TBI, microglial activation rapidly increases the synthesis and release of TNF-α. Studies of human samples have shown that IL-6, TNF-α, and IL-1β mRNA and protein levels increase sharply in the brains of patients who died from TBI [51]. Our previous study also found that TNF-α and IL-1β mRNA and protein levels were significantly increased in rat brain tissue after TBI, accompanied by severe BBB damage and marked neurological deficits [6]. However, delayed early treatment may impair injury repair, aggravate brain injury, and lead to neurological dysfunction [10,11]. In the present study, D-Lim inhibited the protein expression of IL-6, IL-1β, and TNF-α and promoted IL-10 expression, exerting a beneficial anti-inflammatory effect. These findings suggest that D-Lim may be a potential anti-inflammatory therapeutic drug for TBI. This is consistent with Zhang’s research on rhubarb’s treatment of TBI. The study showed that rhubarb can regulate the expression of IL-1β and TNF-α, thereby reducing inflammation, inhibiting cell apoptosis, maintaining the integrity of the BBB, and promoting neural repair [52]. In SH-SY5Y cells, D-Lim significantly downregulated inflammatory factors expression and inhibited apoptosis [53]. Studies in human colon cancer cell models [54], rat liver cirrhosis models [55], and mouse pain models [56] have also demonstrated the favorable anti-inflammatory effects of D-Lim, further confirming its anti-inflammatory activity across different diseases and experimental systems, both in vivo and in vitro.

To further explore the potential effect by which D-Lim regulates inflammation in the treatment of TBI, 29 intersecting genes were identified between the candidate gene of D-Lim and WGCNA-derived genes. Enrichment analysis of these intersecting genes showed that they were mainly involved in the TNF signaling pathway, PI3K-AKT signaling pathway, p38 MAPK signaling pathway, and NF-κB p65 signaling pathway. The candidate genes *Icam1*, *Kdr*, and *Dpp4* were identified through PPI and cytohubba. RT-PCR results indicated that D-Lim could reverse the increase in *Icam1*, *Kdr*, and *Dpp4* mRNA expression caused by TBI. Molecular docking also showed the interactions between D-Lim and these candidate targets. Overall, WGCNA combined with network pharmacology suggested that D-Lim may attenuate TBI-induced inflammatory injury associated with modulation of multiple targets and pathways.

The NF-κB pathway plays a key role in inflammatory responses, particularly by regulating *Icam1* expression and thereby affecting endothelial cell adhesion. Studies have shown that substances such as Salvia and D-Lim reduce *Icam1* expression by inhibiting NF-κB p65 activity, thereby alleviating endothelial inflammation and related pathological conditions [57]. In studies of kidney injury, hydrogen sulfide treatment reduced the concentrations of both NF-κB p65 and *Icam1*, thereby reducing inflammatory renal injury [58]. In the present study, D-Lim treatment after brain injury inhibited phosphorylation of the NF-κB p65 pathway and reduced *Icam1* mRNA expression. This is similar to the study on the intervention of Salvia miltiorrhiza in atherosclerosis [59]. The anti-inflammatory effect of Salvia miltiorrhiza is related to NF-κB and *Icam1* [59]. Therefore, the anti-inflammatory effect of D-Lim may be related to endothelial adhesion molecules. Future studies should further verify this hypothesis and explore its potential therapeutic applications.

NF-κB p65 is a central hub of the inflammatory response and participates in microglial activation, amplification of inflammatory cascades, and neuronal injury; it is regarded as a key signaling axis in neuroinflammation [60]. Activated NF-κB p65 can promote high expression of inflammatory factors such as TNF-α, IL-1β, and IL-6, thereby aggravating neuronal injury [61]. In this study, we found that the NF-κB p65 pathway was activated after TBI, as indicated by enhanced phosphorylation of NF-κB p65, increased expression of Iba1, TNF-α, IL-1β, and IL-6, and decreased protein expression of IL-10. These findings confirmed that a neuroinflammatory cascade was activated after TBI. After Early intervention of D-Lim, NF-κB p65 phosphorylation was inhibited; Iba1, TNF-α, IL-1β, and IL-6 expression was decreased; and IL-10 expression was increased, supporting our hypothesis that D-Lim has favorable anti-inflammatory effects.

The PI3K/AKT signaling pathway is widely recognized as a canonical pro-survival pathway in neurons and is involved in neuronal survival, antiapoptotic regulation, and cellular responses after brain injury [62,63,64]. However, the activation of PI3K/AKT after TBI is not always involved in neuroprotection. In studies on mouse brain injury, it was found that the changes in IGF-1/IGF-1R-AKT were related to the time and location of the injury, and the high expression of p-AKT was observed at the injury site 6 h after brain injury [65]. However, the reduced activation of PI3K/AKT after TBI observed in this study may reflect impairment of endogenous pro-survival signaling, whereas its partial restoration following D-Lim treatment may be associated with attenuation of neuronal injury. Moreover, after D-Lim intervention, AKT phosphorylation increased, whereas NF-κB p65 phosphorylation decreased, suggesting inhibition of NF-κB p65 nuclear translocation and reduced capacity to activate inflammatory cascades, thereby ameliorating neuronal injury. This is consistent with Yang’s research, which indicates that edaravone can alleviate the neuroinflammation mediated by BV-2 microglial cells through the PI3K/AKT/NF-κB pathway [60]. As a downstream factor of PI3K, AKT is phosphorylated following PI3K activation and then regulates downstream substrates involved in inflammatory responses [66]. The PI3K-AKT signaling pathway supports neuronal growth, proliferation, differentiation, and survival in the central nervous system. PI3K phosphorylation activates AKT and thereby exerts neuroprotective effects [67,68,69]. These reports are consistent with our findings: TBI inhibited PI3K activation and AKT phosphorylation, promoted NF-κB p65 phosphorylation, and aggravated inflammatory cascades, which were unfavorable for injury repair. The anti-inflammatory effect of D-Lim may be related to the above-mentioned pathways. This is consistent with findings on Angelica treatment for ischemic brain injury, where Angelica alleviated ischemic stroke-induced brain injury in mice by activating the PI3K/AKT and p38 MAPK pathways [66]. Taken together, studies indicate that TBI-induced neurological injury involves multiple pathways rather than a single pathway. Therefore, we propose that early intervention with D-Lim may regulate TBI-induced inflammatory responses through the combined actions of the PI3K/AKT, p38 MAPK, and NF-κB p65 pathways. This also highlights that the improvement of TBI-induced neurological injury by D-Lim may result from coordinated effects on multiple targets and pathways. Future studies should further explore the potential targets and mechanisms of D-Lim in TBI treatment.

Studies have reported that increased VEGFR2 and VEGF expression after TBI may be protective by promoting angiogenesis, wound healing, and improvement of brain injury [70,71,72]. In this study, the cerebral blood flow, the expression of CD31 and VEGFA were obviously decreased after TBI, whereas cerebral blood flow partially recovered after D-Lim intervention. We speculate that early intervention with D-Lim may reverse vascular injury associated with high VEGFR2 expression during the acute phase of TBI and restore cerebral blood flow, although the specific mechanisms require further investigation.

Finally, we used HE staining to observe the effects of D-Lim treatment on major organs in TBI, and the results showed that D-Lim exhibited good in vivo biosafety. This is consistent with many studies of small-molecule drugs for cerebral ischemia, which emphasize that the prerequisite for treating disease with small-molecule drugs is that they should not harm the body [73,74]. Similarly, ELISA detection of TNF-α, IL-1β, and IL-6 in peripheral blood showed that their expression decreased after D-Lim intervention, indicating that D-Lim has no systemic toxic side effects and exhibits favorable anti-inflammatory activity. These results indicate that D-Lim (10 mg/kg) was safe after TBI 24 h and provide a foundation for subsequent experiments. However, since this assessment was limited to a single dose and a single acute time point, these results should only be interpreted as indicating that no significant acute organ toxicity was observed 24 h after TBI under the experimental conditions of this study. Nevertheless, the exploration of D-Lim toxicity requires further research in the future, which should involve multiple doses, a longer observation period, and specialized toxicological evaluations.

Although D-Lim exerted beneficial anti-inflammatory effects in TBI treatment, was effective in treating TBI, and produced neuroprotective effects by regulating multiple targets and pathways, this study still has several limitations. First, although bioinformatics analysis combined with network pharmacology and molecular docking incorporated multiple lines of evidence, the number of drug and disease targets available in public databases is limited, which may make it difficult to predict all pathways and potential targets through which D-Lim acts on TBI. Second, molecular docking only suggests that the candidate genes identified in this study are related to inflammatory responses, whereas their specific in vivo functions remain unclear. Thirdly, pathway inhibitors were not used in this study, and in vitro experiments were not performed to further validate the mechanism of D-Lim. Therefore, future studies will focus on the predictions generated in this study and further explore the mechanisms of D-Lim through both in vivo and in vitro experiments. Finally, in this study, both pretreatment and post-injury administration were employed to sustain drug concentrations in vivo, with the aim of evaluating the therapeutic effects of D-Lim during the acute phase of TBI. Specifically, we observed that D-Lim exerted anti-inflammatory actions, attenuated neuronal apoptosis, and improved cerebral blood flow in the acute period, thereby providing a theoretical basis for its potential acute protective role in pretreatment or early intervention settings. However, the short observation window precludes definitive conclusions regarding long-term neurorepair; therefore, future investigations should incorporate multiple sampling time points or extend the follow-up period to comprehensively assess its reparative efficacy.

## 4. Materials and Methods

### 4.1. WGCNA

The GSE24047 dataset was downloaded from the Gene Expression Omnibus (GEO) (GEO; https://www.ncbi.nlm.nih.gov/geo/, accessed on 4 April 2026). This dataset contains rat brain transcriptomic profiles from Rattus norvegicus generated on the GPL1355 platform ([Rat230_2] Affymetrix Rat Genome 230 2.0 Array). This dataset included 4 samples from sham group and 12 samples from TBI group. WGCNA (version 1.74) was performed using the WGCNA package in R (version 4.5.1). The optimal soft-thresholding power was screened within the range of 1–30, and a co-expression network conforming to scale-free topology was constructed using a scale-free topology fit index of R^2^ ≥ 0.85 as the criterion. Hierarchical clustering and dynamic tree cutting were then performed based on the topological overlap matrix, with the minimum module size set to 30 genes and deepSplit set to 1.5, to identify co-expression modules. Disease-related key modules were screened by correlation analysis between module eigengenes and the TBI phenotype. Gene significance (GS) and module membership (MM) were calculated, and scatter plots were generated to evaluate the associations of genes with the phenotype and corresponding modules. Genes in the key modules were subsequently extracted.

### 4.2. Acquisition of D-Lim-Related Targets

Potential targets of D-Lim were screened using the Swiss Target Prediction database (https://swisstargetprediction.ch/, accessed on 6 April 2026) and the TargetNet database (http://targetnet.scbdd.com/, accessed on 6 April 2026). The UniProt database (https://www.uniprot.org/, accessed on 6 April 2026) was used to standardize the target names. All targets were then summarized, and duplicate entries were removed.

### 4.3. Mapping of Intersecting Targets

Target mapping was performed using the Venny platform (https://bioinfogp.cnb.csic.es/tools/venny/, accessed on 14 April 2026). A Venn diagram was constructed to identify the intersection between D-Lim-related targets and TBI-related module targets, thereby screening potential therapeutic targets through which D-Lim may regulate TBI.

### 4.4. GO Function Annotation and KEGG Enrichment Analysis

The screened potential targets through which D-Lim may regulate TBI were imported into the Metascape database (https://metascape.org/, accessed on 20 April 2026) for Gene Ontology (GO) functional enrichment analysis and Kyoto Encyclopedia of Genes and Genomes (KEGG) pathway enrichment analysis. The enrichment results were visualized using an online bioinformatics analysis platform (https://www.bioinformatics.com.cn/, accessed on 24 April 2026).

### 4.5. Construction of the Protein–Protein Interaction Network for Intersecting Targets

To further screen and identify candidate targets, the intersecting targets were imported into the STRING database (https://string-db.org/, accessed on 20 April 2026) to analyze protein–protein interaction, and the interaction data were downloaded and exported in TSV format. The data were then imported into Cyto-scape (version 3.7.1) to construct a visual protein–protein interaction (PPI) network. Subsequently, to further identify the candidate gene through which D-Lim alleviates TBI, we screened hub genes using four algorithms (Degree, MCC, MNC, and EPC) in CytoHubba (version 0.1), identifying *Icam1*, *Kdr*, and *Dpp4*.

### 4.6. Molecular Docking Validation

The three-dimensional molecular structure of D-Lim was obtained from the PubChem database PubChem (CID: 440917; https://pubchem.ncbi.nlm.nih.gov/compound/D-Limonene, accessed on 4 April 2026). The crystal structures of the top-ranked candidate target proteins were retrieved and downloaded from the Protein Data Bank (PDB) (RCSB PDB: Homepage, accessed on 4 April 2026). PyMOL (version 2.6.0a0 Open-Source) was used to preprocess the target proteins by removing water molecules and adding hydrogen atoms. Molecular docking between the D-Lim small molecule and the potential target proteins was then performed using AutoDock Vina (version 1.2.5). The conformation with the lowest binding energy was selected as the final docking result.

In order to further explore the potential binding relationships between D-Lim and *Icam1*, *Kdr*, and *Dpp4*. Molecular dynamics (MD) simulations were performed using GROMACS 2024.4 to investigate the binding between D-Lim and three candidate proteins (*Icam1*, *Kdr*, and *Dpp4*). The AMBER14SB force field was used for proteins, while D-Lim parameters were assigned via the GAFF2 force field. To mimic physiological conditions, 30 D-Lim molecules were placed around each protein in a TIP4P explicit water box with a minimum protein box distance of 1.2 nm. The system was neutralized with Na^+^ and Cl^−^ ions at physiological salt concentration. Long-range electrostatic interactions were treated with the particle mesh Ewald method, and van der Waals interactions were computed with a standard cutoff algorithm.

### 4.7. Experimental Animals

Thirty-nine specific pathogen-free male Sprague-Dawley (SD) rats (200–250 g) were purchased from the Department of Laboratory Animal Medicine of Kunming Medical University. The ethical approval number was kmmu20241649 (15 July 2024), and the animal certificate number was SYXK (Dian) K2025-0008. The rats were housed in the animal facility of the School of Basic Medical Sciences, Kunming Medical University, under controlled conditions with standardized room temperature, a 12 h light/dark cycle, and free access to food and water. After a 7-day acclimatization period, the animals were randomly assigned to three experimental groups: Sham group, TBI group, and the TBI + D-Lim group. All rats were euthanized under anesthesia 24 h after surgery for tissue collection.

### 4.8. TBI Model

A rat TBI model was established using the Feeney free-fall method [6,75]. Anesthetized rats were fixed in a stereotaxic apparatus. A circular craniotomy was made at coordinates 2 mm posterior to the coronal suture and 2.5 mm lateral to the right sagittal suture. A 40 g weight dropped from 15 cm caused brain contusion. After hemostasis, the bone window was closed and skin sutured. Rats were kept warm until recovery. Sham rats underwent only anesthesia and craniotomy without impact.

### 4.9. Grouping, Drug Administration, and Specimen Preparation

**Figure 12:**
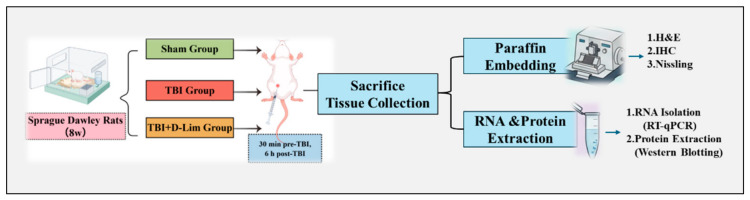
Animal grouping and sample acquisition.

See as Figure 12, after 7 days of adaptive feeding, 39 SD rats were randomly divided into the Sham, TBI, and TBI + D-Lim group (n = 13 for each group). Rats in the TBI + D-Lim group received intraperitoneal injection of D-Lim (10 mg/kg/day) 30 min before model establishment and 6 h after TBI [6,76]. Rats in Sham and TBI groups received an equivalent volume of vehicle. At 24 h after TBI, the rats were anesthetized with 1% sodium pentobarbital [6]. Three rats were randomly selected and fixed with 4% paraformaldehyde for paraffin embedding. The remaining fresh brain tissue samples were used for RNA and protein extraction (Figure 11). Specifically, the sample sizes for each assay were as follows: Western blotting, n = 4 per group; Evans blue staining, Laser speckle imaging, RT-PCR, and histopathological examination (paraffin-embedded sections), n = 3 per group; and ELISA, which was conducted on 3 randomly selected serum samples per group (n = 3). All the animals underwent a modified neurological function impairment scoring test.

### 4.10. Western Blot

Brain tissues from each group were collected, and total protein was extracted using RIPA buffer supplemented with phosphatase and protease inhibitors. Protein concentrations were measured using a BCA protein assay kit. Equal amounts of protein samples (25 µg) were separated by SDS-PAGE and then transferred onto PVDF membranes (Millipore, Billerica, MA, USA). Nonspecific protein binding on the PVDF membranes was blocked by incubation with 5% nonfat milk for 2 h. The membranes were incubated overnight at 4 °C with diluted primary antibodies against TNF-α (Cat. No. 80258-6-RR; 1:1000; Proteintech, Wuhan, China), IL-1β (Cat. No. 16806-1-AP; 1:2000; Proteintech), IL-6 (Cat. No. 21865-1-AP; 1:1500; Proteintech), IL-10 (Cat. No. 60269-1-Ig; 1:2000; Proteintech), p-PI3K (Cat. No. AF3242; 1:2000; Affinity, Changzhou, China), PI3K (Cat. No. AF6241; 1:1000; Affinity), AKT (Cat. No. AF6261; 1:2000; Affinity), p-AKT (Cat. No. AF0016; 1:2000; Affinity), beta-actin (Cat. No. 66009-1-Ig; 1:20000; Proteintech), NF-κB p65 (Cat. No. 80979-1-RR; 1:3000; Proteintech), Iba1 (Cat.NO. GB153502; 1:100; Servicebio, Wuhan, China), CD31 (Cat.NO. GB150217; 1:100; Servicebio), VEGFA (Cat.NO. GB15690; 1:1000; Servicebio), VEGFA (Cat.NO. GB15165; 1:100; Servicebio), and (BCL-2, Cat.NO. EPR17509; 1:3000; Abcam). The next day, the membranes were incubated with HRP-conjugated secondary antibodies at room temperature for 1 h. Protein bands were visualized using a commercial ECL kit (Beyotime Biotechnology, Shanghai, China) and quantified using Image J software 1.54g (National Institutes of Health, Bethesda, MA, USA).

### 4.11. RT-qPCR

Total RNA was extracted from injured cortical tissue using a kit (Cat. No. RC112-01; Vazyme, Nanjing, China), followed by reverse transcription to synthesize cDNA (Cat. No. RR047A, Takara, Kyoto, Japan). RT-qPCR was performed using TB Green (Cat. No. RR820A, Takara, Japan) and specific primers. Relative mRNA expression was calculated by the 2^−ΔΔCt^ method. Primer sequences are listed in Table 4.

### 4.12. Pathological Staining to Observe Histopathological Changes in Brain Tissue

After paraffin embedding, 5 µm sections were prepared. The sections were fixed with 4% paraformaldehyde for 24 h and then subjected to hematoxylin and eosin (HE) staining (Cat. No. G1120; Solarbio, Beijing, China) and Nissl staining (Cat. No. G1430; Solarbio, Beijing, China). Histopathological changes in the injured tissue were observed under a light microscope.

### 4.13. EB Analysis

To examine the leakage of EB in the brain tissue of rats after TBI, the permeability of the BBB was evaluated [6]. At 24 h post-TBI, rats received a tail vein injection of EB solution (2% in saline, 4 mg/kg). After perfusion with saline under pentobarbital anesthesia, brain tissues were collected and the blue staining of the brain tissue was observed.

### 4.14. Data Analysis

Statistical analyses were performed using GraphPad Prism 8.1. Data are presented as mean ± SEM. Group comparisons of single-time-point data (mNSS, histology, Western blot, Evans blue) were conducted by one-way ANOVA with Tukey’s post hoc test. rCBF data across time points were analyzed by two-way repeated-measures ANOVA, followed by Tukey’s test. Normality and variance homogeneity were checked before ANOVA. Statistical significance was set at *p* < 0.05.

## 5. Conclusions

This study combines WGCNA, network pharmacology, and experimental verification to elucidate the potential targets and mechanisms of action of D-Lim in TBI from multiple perspectives. Molecular docking and self-assembling molecular dynamics simulations show that D-Lim may have potential binding affinities with *Icam1*, *Kdr*, and *Dpp4*. Western blot analysis indicates that D-Lim can regulate inflammatory damage caused by TBI, and it is speculated that its anti-inflammatory effect may be associated with the modulation of multiple pathways, mainly including NF-κB p65, PI3K/AKT and p38 MAPK pathways. Western blotting was also used to detect the expression levels of TNF-α, IL-1β, IL-6, IL-10, BAX, and BCL-2. At the same time, immunohistochemical detection of microglial cell activation and vascular injury after TBI was performed, and Evans blue staining was used to observe the permeability of the BBB after TBI. In conclusion, this study preliminarily found that the anti-inflammatory effect of D-Lim may be related to the NF-κB p65, PI3K/AKT and p38 MAPK signaling pathways. Collectively, our data support the potential of D-limonene as an acute protective agent in a pre-treatment/early-intervention paradigm; however, further investigation with clinically relevant post-injury treatment schedules is warranted to determine its translational value. This study provides novel and comprehensive strategies for research and treatment of TBI, and this strategy can also be used to discover new TBI intervention drugs.

## Figures and Tables

**Table 1:** Twenty-nine potential target genes obtained from the intersection of predicted D-Lim targets and WGCNA-derived key modules.

Gene	UniProt	Regulation	Gene	UniProt	Regulation
*Mmel1*	Membrane metallo-endopeptidase-like 1	Up	*Plau*	Urokinase-type plasminogen activator	Up
*Ptpn1*	Tyrosine-protein phosphatase non-receptor type 1	Up	*Map3k8*	Mitogen-activated protein kinase 8	Up
*Ar*	Androgen receptor	Down	*Pim3*	Serine/threonine-protein kinase pim-3	Up
*Ptpn2*	Tyrosine-protein phosphatase non-receptor type 2	Up	*Ptges*	Prostaglandin E synthase	Up
*Rela*	Transcription factor p65	Up	*P2ry2*	P2Y purinoceptor 2	Up
*Itgb3*	Integrin beta-3	Up	*S1pr2*	Sphingosine 1-phosphate receptor 2	Up
*Npy5r*	Neuropeptide Y receptor type 5	Down	*Sele*	E-selectin	Up
*Dpp4*	Dipeptidyl peptidase 4	Up	*Top1*	DNA topoisomerase 1	Up
*Icam1*	Intercellular adhesion molecule 1	Up	*Abcg2*	Broad substrate specificity ATP-binding cassette transporter ABCG2	Down
*Tacr3*	Neuromedin-K receptor	Down	*Srgn*	Serglycin	Up
*Casr*	Extracellular calcium-sensing receptor	Up	*Flt1*	Vascular endothelial growth factor receptor 1	Up
*Fntb*	Protein farnesyltransferase subunit beta	Down	*Ncstn*	Nicastrin	Down
*Hck*	Tyrosine-protein kinase HCK	Up	*Mmp8*	Matrix metallopeptidase 8	Up
*Kdr*	Vascular endothelial growth factor receptor 2	Up	*Shbg*	Sex hormone-binding globulin	Down
*S1pr3*	Sphingosine-1-phosphate receptor 3	Up			

**Table 2:** Potential targets selected from the PPI network.

MCC	Betweenness	Closeness	Degree
*Plau*	*Ar*	*Rela*	*Rela*
*Icam1*	*Rela*	*Icam1*	*Icam1*
*Kdr*	*Icam1*	*Kdr*	*Kdr*
*Dpp4*	*Kdr*	*Dpp4*	*Dpp4*
*Sele*	*Dpp4*	*Sele*	*Ptpn1*
*Flt1*	*S1pr2*	*Flt1*	*Flt1*

**Table 3:** Potential binding energies for molecular docking between D-Lim and *Icam1*, *Kdr*, and *Dpp4*.

Potential Target	UniProt ID	Compound	PubChem CID	Center (X, Y, Z)	Size (X × Y × Z)	Potential Binding Energy (kcal/mol)
*Icam1*	Q00238	D-Lim	440,917	1, −19, 226	88 × 74 × 71	−5.218
*Kdr*	O08775	D-Lim	440,917	−23, −2, −5	56 × 61 × 65	−7.191
*Dpp4*	P14740	D-Lim	440,917	17, −13, 57	96 × 63 × 195	−5.228

**Table 4:** Sequence of Differential Gene Primers.

Gene	Sequence (5′ to 3′)
*Icam1*	F: GGTCAGGGTGCTTTCCTCAA
*Icam1*	R: CTGCTAAAGGTGAGCGTCCA
*Dpp4*	F: GCGCTTGTCACCATCATCAC
*Dpp4*	R: GAAACCCACCGCAAGGAGTA
*Kdr*	F: AGAAGGAACGAGAATGCGGG
*Kdr*	R: CTCTGAAAACGCGGGTCTCT
*Gapdh*	F: ACGGCAAGTTCAACGGCACAG
*Gapdh*	R: GACGCCAGTAGACTCCACGACA

## Data Availability

All the data processing and analysis codes can be provided by the corresponding author upon reasonable request.

## Supplementary Materials

The following supporting information can be downloaded at: https://www.mdpi.com/article/10.3390/ijms27146143/s1.

## Abbreviations

The following abbreviations are used in this manuscript:

D-Lim	D-limonene
TBI	Traumatic brain injury
WGCNA	Weighted gene co-expression network analysis
BBB	Blood–brain barrier
IL-1β	Interleukin-1β
NO	Nitric oxide
TNF-α	Tumor necrosis factor-alpha
TCM	Traditional Chinese medicine
ROS	Reactive oxygen species
DEGs	Differentially expressed genes
GEO	Gene Expression Omnibus
GS	Gene significance
MM	Module membership
GO	Gene Ontology
KEGG	Kyoto Encyclopedia of Genes and Genomes
PPI	Protein–protein interaction
PDB	Protein Data Bank
SD	Sprague-Dawley
BP	Biological process
MF	Molecular function
CC	Cellular component
