# Supplementary material for: Integrated WGCNA and Network Pharmacology Explore the Potential Mechanisms of D-Limonene in Alleviating Traumatic Brain Injury
Source: Int J Mol Sci. 2026 Jul 9;27(14):6143. doi: 10.3390/ijms27146143 (PMC13409895; doi:10.3390/ijms27146143)
Supplement: Supplementary file 1 [file ijms-27-06143-s001.zip › ijms-4381719-supplementary.pdf]

Figure S1.

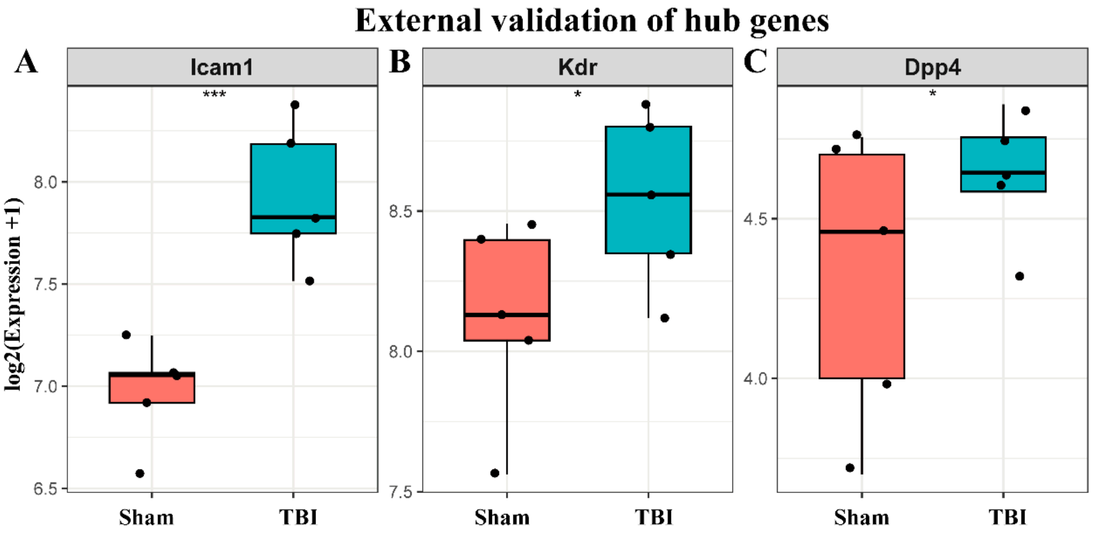

Figure S1. External validation of Hub genes Icam1, Kdr, and Dpp4 in the GSE80174 dataset. The box plot shows the expression levels of the three Hub genes in the Sham group and the TBI group. The vertical axis represents log2 (Expression+1). Compared with the Sham group, the expression levels of Icam1, Kdr, and Dpp4 were all increased in the TBI group, with the difference in Icam1 being the most significant, and Kdr and Dpp4 also reaching statistical significance. The black dots represent individual samples; the box represents the interquartile range, and the middle line represents the median. *$P < 0.05$, ***$P < 0.001$.

Figure S2.

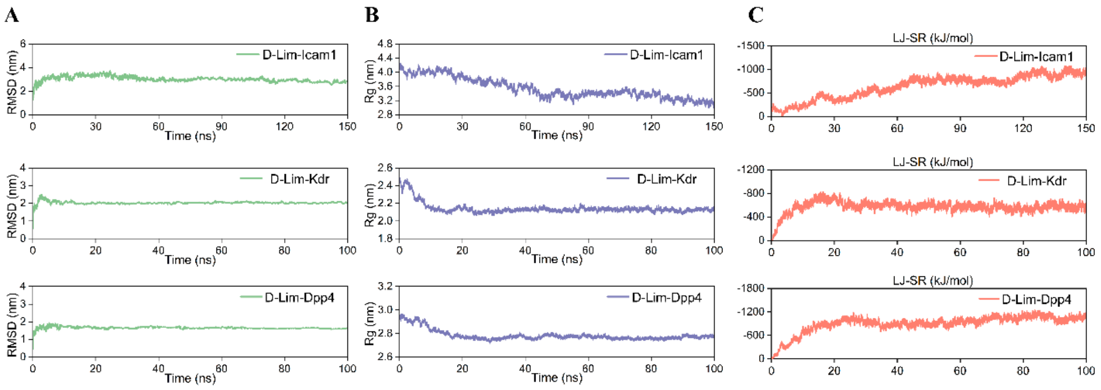

D

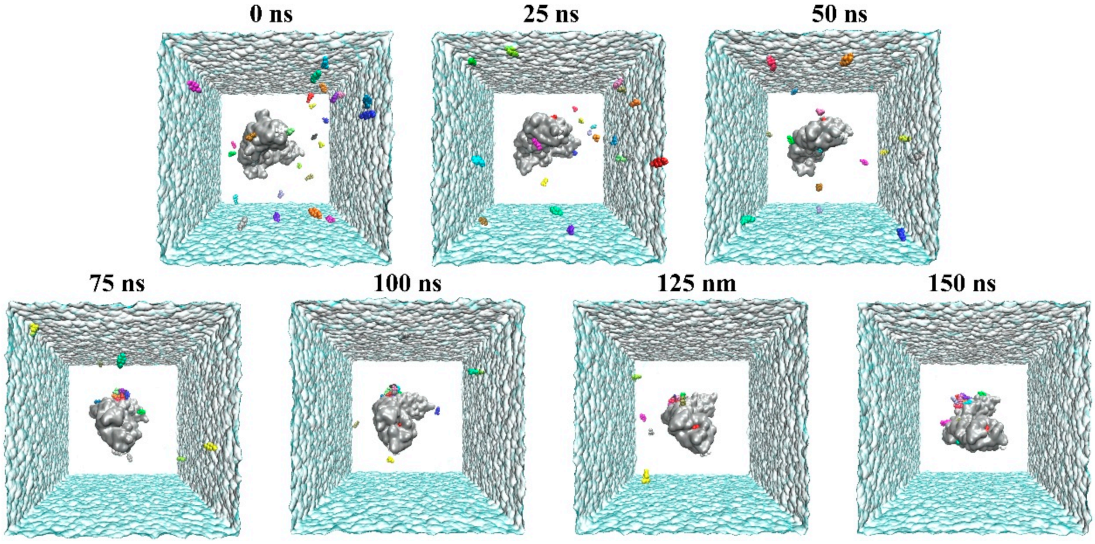

E

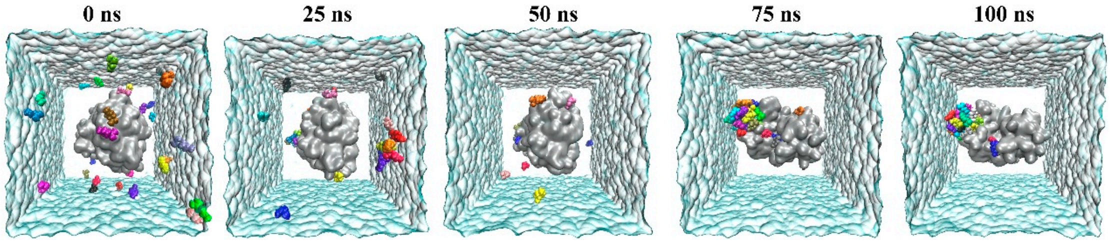

F

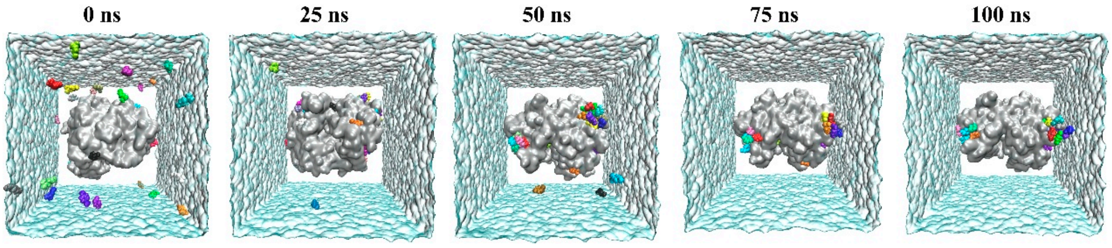

Figure S2. Molecular dynamics simulation predicts the potential binding situation between D-Lim and the candidate protein. (A) RMSD curve reflects the rearrangement process of limonene molecules on the protein surface, from an initial random distribution to a stable adsorption conformation; (B) RG analysis results indicate that the limonene molecule has a tendency to form spatial aggregation in the hydrophobic region of the protein surface; (C) LJ-SR energy analysis indicates that the hydrophobic interactions between the limonene molecule and the surface of Icam1, Kdr, and Dpp4 proteins gradually increase and reach a balance; (D) Snapshots of the binding situation of Icam1-Limonene at five time points of 0, 25, 50, 75, 100, 125, and 150 nanoseconds; (E) Snapshots of the binding situation of Kdr-Limonene at five time points of 0,

25, 50, 75, and 100 nanoseconds; (F) Snapshots of the binding situation of Dpp4-Limonene at five time points of 0, 25, 50, 75, and 100 nanoseconds.
